# “The Window of Opportunity”: A Qualitative Exploration of Individual Reminiscence in Care Home Settings

**DOI:** 10.3390/healthcare14020276

**Published:** 2026-01-21

**Authors:** Aoife Conway, Rosemary Bradley, Assumpta Ryan, Claire McCauley, Brighide Lynch, Deirdre Harkin, Sarah Penney

**Affiliations:** School of Nursing and Paramedic Science, Faculty of Life & Health Sciences, Ulster University, Magee Campus, Northland Road, Derry BT4 87JL, Northern Ireland, UK

**Keywords:** reminiscence, digital health, dementia, person-centred care, care homes, implementation, leadership, qualitative research

## Abstract

**Background:** Care homes are complex care environments where supporting residents’ identity, wellbeing, and sense of personhood is central to person-centred care. Reminiscence is widely recognised as a psychosocial approach that can support these outcomes. However, existing evidence has largely focused on group-based interventions, with comparatively limited attention given to how individual reminiscence is implemented and sustained within care home practice. **Methods:** This study was an implementation-focused qualitative exploration of staff experiences of introducing and embedding individualised reminiscence in care home practice. Care home staff participated in four monthly workshops that introduced principles of individualised reminiscence and supported them to plan and implement reminiscence with at least one resident. Participants used either the InspireD digital reminiscence app (*n =* 19) or non-digital approaches such as life story books (*n =* 2), depending on local preferences and perceived suitability. Three focus groups were conducted with 21 care home staff to explore experiences of implementing individualised reminiscence and perceptions of its impact on residents, staff, and families. Data were analysed using reflexive thematic analysis. **Results:** Four interrelated themes were identified: (1) reminiscence within pressured systems; (2) resident experience and identity; (3) adapting and sustaining practice; and (4) families as partners in reminiscence. Participants described challenges associated with workload pressures, role expectations, and variability in family involvement, which influenced how reminiscence was adopted in practice. Despite these constraints, participants described perceived benefits for residents, including perceived improvements in mood, engagement, and expressions of identity. Participants also discussed perceived increased staff confidence, strengthened staff–resident relationships, and enhanced awareness of person-centred care practices. **Conclusions:** Findings highlight the perceived potential of individualised reminiscence to support person-centred and relational care in care homes, while identifying key contextual influences on implementation. Further research is needed to examine sustainability and effectiveness using comparative and mixed-method designs.

## 1. Introduction

Care homes provide long-term care and support for some of the most vulnerable members of society, including people living with multiple long-term conditions and high levels of dependency. These settings are complex environments, often characterised by workforce pressures, competing priorities, and diverse resident needs [1,2,3]. Within these contexts, maintaining identity, connection, and a sense of personhood is central to residents’ quality of life [4,5]. To support this, international policy and practice guidelines advocate for care practices tailored to individual preferences and life histories [6,7].

Reminiscence is one such approach, encompassing a range of psychosocial practices that draw on personal memories to stimulate communication, reflection, and connection, with the aim of supporting wellbeing [8]. Research has explored the use of reminiscence across a range of populations and settings, including dementia care [9,10,11], people who have experienced a stroke [12,13], mental health and mood-related interventions [14], Parkinson’s disease [15], broader ageing [16], and intellectual disability [17].

A Cochrane review [9] explored the effects of reminiscence therapy for people living with dementia and their carers and reported that one-to-one reminiscence using individualised memorabilia demonstrated the strongest empirical evidence for improving communication and interaction with probable benefits for cognition and mood. Within care homes, individualised reminiscence has particular psychosocial relevance, where residents may experience a loss in personal identity, reduced opportunities for self-expression, and a change in social roles [18]. Although there is a growing evidence base examining the effectiveness of reminiscence interventions in care homes, considerably less attention has been paid to questions of implementation, sustainability, and the mechanisms of action through which reminiscence is understood, adapted, and embedded within everyday care home practice. Existing studies have predominantly focused on pre–post outcomes or researcher-led delivery, offering limited insight into how staff engage with, operationalise, and maintain individualised reminiscence within this organisational context [3,9,19,20]. Where such studies exist, they have predominantly examined group-based interventions designed to stimulate social or cognitive interactions [21].

This project was undertaken as part of the wider My Home Life programme, a leadership support programme that aims to enhance the quality of life for all those who live, work, die, and visit care homes (https://www.myhomelifeni.co.uk) (accessed on 15 December 2025). The programme is currently being rolled out across care homes in a region in the UK and is specifically targeted at staff who hold leadership roles within their organisations [22]. It typically attracts senior managers, established leaders, and aspiring leaders seeking to further develop their leadership skills. As part of the programme in this region, participating care home staff are invited to consider the introduction of individual reminiscence within their care homes.

In the current study, individualised reminiscence was supported through resources that enabled staff to curate and share meaningful personal materials. This included access to the InspireD reminiscence app (www.InspireD.com) (accessed on 15 December 2025), a tool originally co-designed with people living with dementia and their carers to support the creation and use of individualised memorabilia. Within this study, the app functioned as a practical resource to support staff in facilitating individualised reminiscence, alongside non-digital approaches such as life story books. Research to date on the InspireD reminiscence app has focused on home-dwelling dyads, reporting positive user experiences and describing experiences of individualised, memory-based interaction in supporting engagement and communication [23,24]. Its availability within this project offered staff an additional way to support individualised reminiscence alongside the resources already used in practice.

### Aim

This study aimed to explore staff experiences of implementing individualised reminiscence and to explore participants’ perceptions of impact within care home settings.

Objectives:

(1) To explore participants’ experiences of implementing individualised reminiscence within everyday care home practice.
(2) To examine participants’ perceptions of the impact of individual reminiscence intervention on residents, staff, and families.
(3) To identify participants’ views on the opportunities and challenges associated with implementing individualised reminiscence in care homes.

## 2. Materials and Methods

### 2.1. Study Design

This study adopted an exploratory qualitative approach to meet the aims and objectives. Qualitative data were gathered through focus group interviews (*n =* 3) with care home staff (*n =* 21) to capture their experiences, insights, and reflections. Ethical approval was obtained from the participating university where the researchers were based (REF: FCNUR-21-039). The Consolidated Criteria for Reporting Qualitative Research (COREQ) checklist [25] informed the design, conduct, and reporting of this study.

### 2.2. Recruitment and Participants

A convenience sampling approach was adopted. Recruitment was facilitated through the My Home Life programme (https://www.myhomelifeni.co.uk) (accessed on 23 December 2025). Participants were eligible for inclusion if they were (1) care home staff employed in organisations participating in the My Home Life programme within the study region, (2) involved in supporting, leading, or facilitating its implementation within their care home, and (3) able and willing to provide written informed consent. Participants were excluded if they did not meet the inclusion criteria or did not consent to take part in the focus group data collection.

Twenty-nine staff engaged with the programme component focused on individualised reminiscence and were involved in its implementation within their care homes. Of these, 21 provided informed consent to participate in the research study. Participants held roles that enabled participation in the programme, including managerial, leadership, or senior clinical positions. Findings therefore reflect perspectives from these positions, which may influence transferability to care staff in other contexts.

### 2.3. Intervention

Participants engaged in four workshops focused on individualised reminiscence, delivered monthly over four months. The workshops introduced the principles of individual reminiscence, supported participants to facilitate and embed the approach within their teams, and encouraged reflection on early experiences of use in practice. Participants were supported to integrate individualised reminiscence within their care homes using either the InspireD reminiscence app or non-digital resources such as life story books. Where the InspireD app was used, a dedicated tablet device was provided to support implementation.

Participants identified at least one resident and worked collaboratively with staff and family members to select meaningful memories or prompts for inclusion. They then trialled reminiscence interactions and supported colleagues to facilitate these interactions as part of routine care. Participants were instructed to implement individualised reminiscence in line with core principles introduced during the workshops, including the use of personalised materials, prioritising one-to-one engagement where possible, responsiveness to residents’ preferences and emotional cues, and integration into everyday care interactions. No fixed schedule, frequency, or duration of reminiscence sessions was prescribed; instead, participants were encouraged to apply the approach flexibly in response to residents’ needs and the realities of care home practice. The memorabilia used was determined entirely by residents and families, with no guidance or criteria imposed by the research team. The research team did not access, review, or extract any content from the InspireD app or other reminiscence materials.

The majority of participants (*n =* 19) implemented individualised reminiscence using the InspireD app, with a small number (*n =* 2) using non-digital resources such as life story books. The study was not designed to compare these approaches.

### 2.4. Data Collection

Data were collected during three focus group interviews (June 2025). An interview topic guide was used to prompt discussion aligned with the aims and objectives of the study.

The focus group topic guide was developed by the research team and informed by the study aims and objectives, the relevant literature on reminiscence and implementation in care home settings, and the content of the My Home Life programme. The guide was reviewed by the research team prior to data collection to ensure clarity, relevance, and appropriateness for the context. It was piloted with a small group of My Home Life participants who were not involved in this study, and minor refinements were made to the wording and sequencing of questions.

Focus groups were conducted in person (6–8 people per focus group) and lasted 60–90 min. All interviews were audio-recorded with participant consent and transcribed verbatim. Identifying details were removed to preserve confidentiality. Written informed consent was obtained from all participants prior to data collection, including consent for the use of anonymised data in dissemination and publication.

### 2.5. Data Analysis

Data were analysed using reflexive thematic analysis, following the six-phase approach outlined by Braun and Clarke [26]. Analysis moved beyond descriptive summarising through iterative engagement with the data. Initial codes captured explicit content in participants’ accounts, but were subsequently examined for patterns, underlying meanings, and relationships across the dataset. Through team-based reflexive discussion and memo writing, codes were interrogated in relation to the study aims and refined into themes that offered interpretative explanations of how individualised reminiscence was experienced within care home contexts. Themes were developed to capture shared patterns of meaning, rather than to catalogue the frequency of responses, and were refined through repeated movement between the data, codes, and emerging thematic structure [26].

The research team was involved in the facilitation of My Home Life QI sessions and/or had prior involvement in the development and evaluation of the InspireD reminiscence app. This positionality provided contextual insight but required continual reflexive awareness of potential bias. Transcripts were shared within the research team to support reflexivity and critical interpretation and to explore potential blind spots and alternative interpretations, rather than to achieve coding consensus. Participant quotes are used in the Findings section to illustrate each theme and preserve the authenticity of their perspectives.

## 3. Results

Participant characteristics are summarised in Table 1. These participants were working across 21 care homes situated throughout a region within the United Kingdom. The care homes varied in size, accommodating between 15 and 76 residents, were located in both urban and rural settings, and included both nursing and residential facilities catering to people with diverse care needs. Across the 21 participants, 2 decided to use life story books, while the remaining participants chose the InspireD reminiscence app to support individualised reminiscence. The findings therefore predominantly reflect the staff’s experiences of implementing digital reminiscence, although themes relating to implementation processes were shared across both digital and non-digital contexts.

Through reflexive thematic analysis, four interconnected themes were generated that capture how reminiscence was experienced and embedded in care homes (Table 2). Across themes, references to changes in residents’ mood, behaviour, or care-related outcomes reflect staff-reported observations and interpretations rather than objectively measured effects.

### 3.1. Theme 1: Reminiscence in Pressured Systems

Participants revealed that the introduction of individualised reminiscence was initially met with reluctance from staff and perceived as a disruption to existing routines and workloads. Hesitation was not about reminiscence itself, but about how it would fit into already pressurised systems of care, given the individual nature of the implementation:

“*I did come across quite a lot of what’s this about, where will I get the time, why am I only doing this with one resident, how did you choose that one resident, I was hearing a lot of that at the start… a lot of what is she doing now.*”

(Participant 14)

This sense of scepticism was framed by others as part of a predictable cycle of resistance to change and many of the participants felt that reluctance stemmed less from disinterest in reminiscence and more from uncertainty of something new:

“*I think it was my expectation because any time you’re going to adopt change you get that resistance, that’s the first wave, we’re used to working through things like that.*”

(Participant 3)

“*For my staff anyway, it was probably just the unknown, this was something different.*”

(Participant 7)

Time pressures were consistently the most discussed barrier. Staff framed individual reminiscence as an additional “task” layered onto their existing duties:

“*At the start the reaction was very much, ‘We don’t have time for this.*”

(Participant 11)

The participants acknowledged these concerns as valid, given the realities of staffing levels:

“*For my staff it was really hard to find the time, because generally during the day you have two people on the floor, so it was hard to find time for one-to-one.*”

(Participant 5)

The difficulty was especially evident as this project focused on individual reminiscence targeted at one resident:

“*But it was a big difficulty in just trying to encourage a staff member that this is the right way, just because of the limits with the time management and having to spend a wee bit more time with the specific resident, obviously being taken away from other duties.*”

(Participant 16)

Over time, some participants adapted the approach by shifting from individual reminiscence to small-group delivery, which they perceived as a more workable fit within existing routines. This was perceived as a more efficient use of staff time. However, this adaptation highlights a tension between individual reminiscence, which depends on individually tailored content, and operational pressures that favour group delivery, with the potential to dilute the individualised focus central to individual reminiscence:

“*At the beginning they were a bit hesitant… but now they make time, more for groups or maybe small groups, but one-to-ones, there’s just not enough time really for one-to-ones.*”

(Participant 9)

“*We thought maybe doing it as a group would be more effective, for example, showing the photos of one resident and letting other residents join the conversation. That might work better.*”

(Participant 3)

Role perceptions further complicated implementation. Participants explained that carers often viewed reminiscence as the remit of activity staff rather than their own responsibility; however, for some, this changed throughout the project:

“*We have three nearly activity staff but covering two full time roles, the carers and the other staff don’t feel like it’s their role… they’ll have those chats but I couldn’t see a carer going and picking up the iPad to go and do it but the activity staff will.*”

(Participant 4)

“*I felt before the activities person was always doing that role but now the staff are more involved in it since this programme*”

(Participant 20)

Existing routines also influenced what was real and possible; this included the routine of staff but also the routines of residents. Participants emphasised the importance of “*catching the moment*” (Participant 3) within these routines:

“*You might be ready to do it, but they’re not in the form for it, and if you miss that window, it’s gone until another time.*”

(Participant 18)

Key drivers or champions within the team often acted as catalysts for implementation and explained how one enthusiastic individual could make a shift within the team:

“*I was lucky in that one of the care assistants… is just really engaging and so, so interested so she took it and went with it*“

(Participant 10)

As staff became more familiar with individual reminiscence, some described reminiscence as less demanding and more effective in supporting residents’ perceived wellbeing. Staff began to see the individualised approach to reminiscence as manageable and even beneficial, with some reframing reminiscence as a time-saver:

“*I think soon after everything runs quickly and smoothly then staff soon realise that it freed up more time because there were less distress reactions… actually, they were able to manage behaviours better.*”

(Participant 9)

### 3.2. Theme 2: Resident Experience and Identity

This theme illustrates participant perceptions of how individual reminiscence was experienced by residents as a way of reconnecting with self, relationships, and community. The most commonly reported perceived change was improved mood and positive engagement. One participant described a resident who had previously been isolated and distressed, but began to participate more fully in home life:

“*This resident had not been involved in any activities before, displayed very challenging behaviour, and required one-to-one supervision, rarely leaving her room. With the reminiscence sessions, she slowly started coming out of her room, engaging in activities, and mingling with other residents… Staff feel her improvement has been dramatic, and we plan to introduce the app slowly to other residents to see how it works for them.*”

(Participant 7)

Participants reported observing changes in residents’ affect and engagement during reminiscence interactions, which they interpreted as positive responses: “You’re giving them the undivided attention and it’s pure benefits the whole way from there.” (Participant 21)

“*Once you start that one wee song on the app, the tablet gets put down because she’s up doing something, and that totally changes her mood.*”

(Participant 3)

Participants described how reminiscence could prompt embodied responses that reconnected residents with familiar roles, skills, and aspects of self. One staff member recalled a secretary who lit up at the sound of a typewriter; this response was interpreted as a momentary re-engagement with a valued occupational identity, demonstrating how sensory cues can evoke embodied memory and personal meaning:

“*The sound of a typewriter … when she heard it the first time her whole face just completely lit up and she started tapping.*“

(Participant 11)

Participants interpreted reminiscence as reinforcing residents’ sense of self and continuity of identity, allowing residents to affirm aspects of who they are beyond a diagnosis. Pride in personal appearance also emerged as an expression of identity, particularly as residents responded to photographs:

“*Overall, it’s been good to see… it’s had a bit of an impact on him because he has that image and linked identity*.”

(Participant 19)

“*You could see she still has that sense of identity… it gives them that recognition of themselves.*”

(Participant 16)

Participants explained how getting to know residents at a deeper level was perceived to support aspects of everyday care, such as encouraging fluid intake.

“*We found out she actually likes wee teapots… and she was drinking from a teapot with no problem, and we can’t really get her to drink otherwise. Her volume of fluids [pause] they have increased.*”

(Participant 8)

The focus on reminiscence within the care home also appeared to foster social interaction and community belonging. Previously withdrawn residents began opening doors, joining activities, and forming friendships:

“*And he’s even teaching me to play chess, and he’s learned other residents to play chess. He usually would stay in his room more, but now he has the door open and, you know, whereas before he never would have done that as much. He’s opened up more, you know*”

(Participant 12)

“*My activities coordinator combined current and past pictures of her, and that really encouraged her to participate in activities and come out of her room. I’ve been able to capture moments showing how she has come out of herself.*”

(Participant 20)

Staff interpreted these changes as strengthening relationships within the care home and enhancing its sense of community:

“*You can just show through the relationships that people have built up are stronger, and the community spirit is stronger.*”

(Participant 14)

Engaging in individual reminiscence was seen as something that “*builds trust and security*” (Participant 21) between staff and residents. Participants described how one-to-one reminiscence appeared to contribute to deepened relationships and fostered more meaningful connections:

“*Some of them, the one that I’m doing it with, they enjoy it more, it’s more engagement, conversations, but he’s more approachable. He’s always joking now with me.*”

(Participant 1)

Some participants saw how reminiscence was not only an enjoyable activity but also a meaningful therapeutic intervention, which staff perceived as supporting residents in coping with loss or regulating emotions:

“*Our resident is grieving, she lost her husband… using it at the appropriate time can be really mood changing, it can lift her mood.*”

(Participant 10)

However, for some residents, reminiscence risked triggering distressing emotions, and staff described how certain memories could be upsetting, suggesting that sensitivity to each resident’s preferences, and emotional responses is central to delivering person-centred reminiscence within care home settings:

“*Bringing up different things about her life upsets her. When you show her pictures of her family or herself in the past, she will laugh at first, but then it turns into a hysterical laugh and then she starts to cry.*”

(Participant 2)

Participants discussed the importance of finding the “right memory.” Participant 8 highlighted the individual nature of reminiscence, and suggested that meaningful engagement depends on identifying personally resonant memories rather than applying a uniform approach:

“*Reminiscence can’t be forced; it’s about the right memory for the right person. For some, certain memories make them agitated. One resident I thought I’d start with doesn’t like seeing herself, even in a mirror. She’ll turn away. But if you show her a photo of someone else, she’s delighted—she’ll laugh, especially if it’s someone she likes. It works in different ways for different people.*”

(Participant 8)

While some residents responded positively when reminiscence was carefully tailored to their preferences, others appeared less engaged. Participants suggested that not all individuals find meaning through reminiscence itself. For some, alternative forms of connection and meaningful connection were more effective:

“*It became less of an activity and more of a task. I think she would have enjoyed more just the general conversation, doing artwork, or going for a walk, rather than the activities therapist trying to input all this data, because it didn’t really resonate back with her. So, in that way, it wasn’t an activity, it was a task.*”

(Participant 15)

### 3.3. Theme 3: Adapting and Sustaining Practice in Care Homes

This theme illustrates how participation in the reminiscence project heightened staff awareness of reminiscence as a purposeful and relational practice and became more valued within everyday care:

“*It has triggered all the staff to realise that reminiscing is quite an important factor.*”

(Participant 11)

Early integration at admission was seen as crucial. Participants emphasised that reminiscence should not be added later as an optional activity but woven into care planning from the outset. Staff described admission as a “*window of opportunity*” (Participant 12) for embedding reminiscence. This positioning of reminiscence as part of the admissions process framed it as integral to knowing the person, not an optional activity.

“*If it’s set up in the first few weeks of admission, alongside the life story, that’s really the hardest work taken out of it.*”

(Participant 19)

“*If it’s started at admission and integrated into the nursing team, it tends to filter down and become part of practice, not an add-on.*”

(Participant 18)

Throughout the project, participants recognised how reminiscence supported a broader range of residents beyond residents living with dementia or those with higher levels of cognitive or physical needs:

“*And I suppose it not just for those residents that have a diagnosis of dementia. I could see it being beneficial to other residents.*”

(Participant 6)

“*My perspective has totally changed. At first, I thought it should be somebody who was more capable and active to put things into it, but I now think it would be a lot more beneficial for a resident who can’t.*”

(Participant 3)

Participants reported that this raised awareness translated into changes in everyday interactions. Rather than focusing narrowly on routine care tasks, reminiscence encouraged them to bring residents’ histories into daily contact opportunities and staff appeared to be more curious about residents:

“*You do see us interacting more with them, bringing up more about their lives, not just like taking them to the bathroom or doing those things all the time.*”

(Participant 4)

“*I think it’s making my team of staff more curious about the other residents, finding out more about their background… it’s making them think oh gosh, what did they do?*”

(Participant 2)

For some, this knowledge about the resident’s life story became as essential as clinical history for providing care:

“*It’s something that you’re doing every day, and it probably is one of the most important things you need to know. just as much as all the medical history.*”

(Participant 7)

“*If we didn’t have residents’ life stories to reminisce on, I would find it very difficult, what are we actually going to talk about?*”

(Participant 12)

Participants described how engaging with individual narratives enabled staff to adopt more person-centred approaches, and increased confidence in responding to distress, becoming less reliant on medication and more skilled in using reminiscence to de-escalate distress or agitation:

“*Before they might have got quite tearful and distressed themselves when a resident was very agitated… then the next thing, you’d see someone sitting laughing beside her and she’s calm again. You can see they’ve done something other than reaching straight for medication, and it’s worked.*”

(Participant 16)

Participants also explained that by engaging in individual reminiscence, staff reported that they saw residents differently and displayed more empathy:

“*An increased connection with the resident, and empathy as well… the resident expressed actually how more value they feel, that their time isn’t just about right, up, dressed, you know, it’s about it being more meaningful.*”

(Participant 20)

Reminiscence also prompted staff to rethink the category of “activities.” And some could see the value of reminiscence being integrated fluidly into daily life rather than planned as rigid tasks.

“*Even the word ‘activities’… If I think of my granny at home doing activities, it would never be called that. It’s just normal daily things she would have done at home.*”

(Participant 13)

“*Instead of having a rigid ‘men’s chat’ or ‘ladies’ tea’… you can have something that sparks conversation naturally.*”

(Participant 18)

For staff themselves, individual reminiscence gave their work new meaning and contributed to role satisfaction:

“*I think it actually gives the staff a purpose as well, because they are recognising that this is an individual who has had a whole life and a life story, rather than just ‘this is a task, and I do a job.’ If they’re able to engage with them and be more personal, they’re treating them and respecting them as they should, rather than ‘this is my job, I do this, this, and this.’ I always say to the staff, enjoy them. Because if you don’t do that, how do you get enjoyment in the job?*”

(Participant 21)

### 3.4. Theme 4: Families as Partners in Reminiscence

This theme captures both the enabling and limiting roles families played in reminiscence, showing how collaboration could be transformative, but absence or emotional strain could act as barriers.

From the outset, participants stressed the importance of family buy-in. Relatives who were engaged contributed photographs, videos, and sounds (memorabilia) that enriched the reminiscence experience and made the process straightforward:

“*The family members were very, very involved, always very involved in the care and very interested. So, all of that made it, I suppose, a positive experience from the outcome.*”

(Participant 1)

Where relatives were highly engaged, this enhanced the continuity of reminiscence but also created shared ownership of the process, easing the pressure on staff while deepening collaboration between families and staff:

“*Because the family member got so involved… staff’s involvement didn’t have to be so intense then, because the next of kin took it on quite well with her mummy. And then from that the staff could see the benefits; it was like a ball rolling.*”

(Participant 11)

Other participants acknowledged that family contributions were not always forthcoming. Without family input, reminiscence was difficult to sustain:

“*Ultimately if you don’t have photos and enough information, you’re not going to get the full benefit.*”

(Participant 8)

Some families were simply disinterested because they viewed it as a task, and others viewed it as staff’s responsibility.

“*Families weren’t interested either, they tried it a couple of times, they said no, can’t be bothered with that.*”

(Participant 17)

Family dynamics could also prevent access to life stories and associated memorabilia for some residents:

“*There was a dynamic in the family that she couldn’t really provide a photograph from years ago because there’s a split in the family and they couldn’t get anything of her. So, I stopped asking then.*”

(Participant 6)

In other cases, the “nearest” family member was distant, and some residents had no relatives at all:

“*Sometimes the next of kin could be a cousin rather than a husband, sister or brother and they don’t really have that much information.*”

(Participant 12)

“*Some residents don’t have relatives or are abandoned by family. Sometimes it’s very hard to collect life history, so it may not work for them.*”

(Participant 14)

These examples made participants uneasy about the equity of access. They worried that residents without family involvement might be excluded from reminiscence altogether:

“*Well, we were saying there if somebody didn’t have a family would they be able to do it? Because family involvement is so important, I don’t know.*”

(Participant 2)

Families could also struggle with the emotional weight of reminiscence. For some, watching a loved one fail to remember was distressing and led them to disengage:

“*In my case, initially it was very good, but whenever he couldn’t remember them, it really hurt them… now… they don’t want to even show them. The first week was very good, but then it just stopped.*”

(Participant 3)

Some staff approached reminiscence cautiously, worried that introducing it might raise expectations or inadvertently cause distress for families. Participants felt gaining family buy-in required sensitivity; enthusiasm needed to be balanced with a careful management of expectations:

“*The reason why then I didn’t choose to go with it because I thought you know what, I think it actually could be maybe more upsetting for her husband if she didn’t engage and he was maybe quite excited or thinking this might work or this might help, and that’s why I didn’t but that’s where I wanted it because I thought that might help her and be beneficial for her.*”

(Participant 15)

Despite these challenges, participants perceived that families themselves benefitted from reminiscence, particularly through observing changes in residents and the opening of new conversations between relatives and their loved ones:

“*They’ve seen the benefits of it from their father having less episodes of being distressed…So, from an outcome point of view that’s less distressing for them as relatives as well*”

(Participant 20)

Participants felt that it gave families reassurance that staff wanted to know their relatives more deeply and opened conversations between staff and family members, creating shared discovery and trust:

“*Like family members are coming in and I think it gives them a confidence that we truly want to get to know their relative, it instils a sort of confidence in them that their loved one is being cared for on a more meaningful level than just ticking boxes. I think it makes it more meaningful.*”

(Participant 7)

“*It was lovely probably for them to hear that we were doing this… it opens conversations… they’re very much engaged with the fact that you’re interested.*”

(Participant 10)

## 4. Discussion

This qualitative study explored care home staff experiences of implementing individualised reminiscence in the care home setting and their perceptions of impact. It begins to address gaps identified within the existing literature, particularly, the limited evidence on the implementation of reminiscence in care homes, as highlighted by previous authors [3,9,19,20]. These findings foreground reminiscence not as diversionary activity but as relational work that supports the continuity of identity, belonging, and social participation within the care home context.

Participants described both the promise and the practical realities of implementing individualised reminiscence within busy services, offering valuable insights and raising important questions about how reminiscence can be sustained as part of everyday care. The findings demonstrate that reminiscence was understood in varying ways, ranging from being viewed as a discrete “activity” to being valued as an integral component of everyday care that informs assessment, care planning, and daily interactions.

Importantly, the data reveal a feasibility gap between the aspiration to deliver individualised, one-to-one reminiscence and the operational realities of care home staffing and workload. In practice, implementation was frequently constrained by time, competing priorities, and role expectations, leading to adaptations such as small-group delivery. This has implications for scalability; wider roll-out is unlikely to be achieved through individual motivation alone and may require explicit resourcing, including protected staff time, workforce development and supervision, and organisational processes that embed reminiscence within routine care [27,28].

Group sessions offered operational advantages whilst the individualised and meaningful nature of individual reminiscence was highly valued. This raises an important question about whether reminiscence must be treated as a binary choice. Several participants suggested a potential middle ground by incorporating individualised materials within group contexts, although this would require careful attention to issues such as consent and confidentiality. This hybrid approach may warrant further exploration, as it may offer a way to retain the depth of individualised reminiscence while benefiting from the feasibility of group delivery. This echoes previous research highlighting the differences in how reminiscence is conceptualised and operationalised within care settings [11,20]. However, the present study extends this evidence by highlighting the participants’ views that reminiscence should be systematically embedded within organisational processes, particularly during admission and review stages, so that residents’ life histories are routinely captured, updated, and actively used to inform person-centred care planning. Treating reminiscence as an integrated component of care, rather than an optional activity, could be a practical step that embeds person-centred care in everyday practice [5,29]. From an implementation perspective, this also raises important questions about fairness and access, particularly in care homes where some residents have limited or no family involvement. Without intentional organisational strategies to support residents who lack family advocates, individualised reminiscence may inadvertently privilege those with stronger social networks.

Participants also emphasised that staff engagement was central to embedding reminiscence and discussed issues with initial buy-in. Findings point to barriers such as role clarity, time and workload, all of which must be addressed if reminiscence is to become routine practice [3]. In some cases, attitudes shifted when staff observed direct benefits for residents and relationships (however, this was not always the case). Participants identified role models and training as key drivers that facilitated change in practice. These findings suggest building staff capability and confidence in delivering individualised reminiscence may be worthwhile.

While reminiscence was frequently perceived as meaningful and beneficial, the findings also highlight potential emotional risks. Participants described occasions where reminiscence elicited distress, grief, or overwhelming emotion for residents and, at times, for family members. These accounts reinforce the importance of skilled facilitation and emotional sensitivity, particularly when working with deeply personal or loss-related memories [8,9]. Individualised reminiscence should therefore not be assumed to be universally positive; rather, it requires ongoing assessment, flexibility, and the capacity to pause or redirect when emotional responses indicate that reminiscence may not be appropriate at a given time. In light of these findings, education and training should focus on supporting staff to respond effectively when residents become distressed, fostering confidence in the use of reminiscence, empowering and recognising key drivers within teams, and engaging and supporting families as active partners in the process. Embedding reminiscence into workforce development frameworks may strengthen sustainability and ensure greater consistency across care homes [30].

The variability in responses regarding buy-in, implementation, and perceived success of the project reflects what the updated Medical Research Council (MRC) framework for developing and evaluating complex interventions identifies as critical for successful implementation: recognising that change is non-linear, context-dependent, and influenced by underlying mechanisms [31]. Future research on embedding reminiscence therefore requires not only outcome evaluation, but also close attention to the contexts and mechanisms that contribute to successful and sustainable implementation [31]. Future research could examine implementation across multiple care providers, incorporate the perspectives of residents, families, and frontline staff, and where possible, evaluate outcomes alongside experiences.

## 5. Conclusions

This study provides insights into staff experiences of implementing individualised reminiscence and their perceptions of its impact within care home settings. Participants described perceived changes in residents’ mood and engagement, strengthened relationships, and increased person-centred practices. Implementation was variable and depended on context, with time pressures, role clarity, and family engagement acting as key constraints, while key drivers and practical, ongoing training were suggested to be important for uptake. Embedding reminiscence within admission and review processes, workforce development, and flexible delivery modes may support sustainability. Further research is needed to explore how individualised reminiscence can be implemented equitably and sustained across diverse care home contexts, and to examine how training, leadership, and family partnership shape implementation processes.

## 6. Strengths and Limitations

This study has several strengths, including capturing views and experiences across a wide geographical area and 21 diverse care home settings. The imbalance between digital (*n =* 19) and non-digital (*n =* 2) approaches limits any meaningful comparison between these modes of delivery. While the study was designed to explore implementation processes rather than to compare tools, the findings primarily reflect staff experiences of implementing the InspireD digital reminiscence app. Caution is therefore required when interpreting references to non-digital approaches, and further research is needed to examine the implementation of individualised reminiscence using non-digital methods in greater depth. The use of convenience sampling through a leadership development programme resulted in a sample predominantly comprised of managers and senior clinical staff. While this provided valuable insight into implementation and organisational processes, it also represents a systematically partial perspective, with limited representation of other care staff, residents, or family members. As such, findings are most transferable to understanding implementation from a leadership and service-development standpoint, rather than reflecting the full range of experiences involved in delivering individualised reminiscence in everyday care. Rigour was supported using COREQ guidance, reflexive thematic analysis, and team reflexivity, with findings illustrated by rich quotations that strengthen transferability. Researchers also delivered the programme and led the roll-out of the project, which may have influenced responses and introduced both social desirability and analytic biases. However, several steps (as outlined above) were taken to enhance rigour and minimise these risks. The process by which staff selected the individuals who participated in the individualised reminiscence may have influenced which residents were deemed suitable or likely to benefit, thereby shaping the nature of the data generated. No information was collected about residents’ demographic characteristics, care needs, or cognitive status, as the study focused on staff experiences of implementation rather than resident outcomes. However, the absence of this contextual information is a limitation, as these factors may influence how individualised reminiscence is used and experienced within care homes.

## Figures and Tables

**Table 1:** Participant characteristics.

		*n=*
Participant role	Care Home Manager	11
	Acting or Deputy Manager	3
	Senior nursing staff (Charge Nurse/Ward Sister/Senior Staff Nurse)	3
	Staff Nurse	4
Years working in care homes	1–3 years	2
	3–5 years	1
	5–10 years	5
	11–15 years	4
	16–20 years	3
	>20 years	6

**Table 2:** Overview of themes.

Theme	Theme Focus	Key Analytic Findings
Theme 1: Reminiscence in pressured systems	How organisational pressures shape feasibility, staff buy-in, and adaptation of individualised reminiscence	Time constraints, workload, role boundaries, adaptation to small-group formats
Theme 2: Resident experience and identity	Staff perceptions of how individualised reminiscence supports identity, engagement, and emotional connection	Mood and engagement, embodied responses, identity affirmation, emotional risks
Theme 3: Adapting and sustaining practice	How reminiscence is normalised, embedded, and sustained within everyday care	Integration into admission processes, leadership, staff confidence, reframing reminiscence
Theme 4: Families as partners in reminiscence	Opportunities and challenges in family involvement with life histories and reminiscence	Family involvement, equity of access, emotional burden, partnership dynamics

## Data Availability

Data available on request from the authors to avoid public disclosure of private data.
